# The association of psychiatric comorbidity and use of the emergency department among persons with substance use disorders: an observational cohort study

**DOI:** 10.1186/1471-227X-8-17

**Published:** 2008-12-03

**Authors:** Geoffrey M Curran, Greer Sullivan, Keith Williams, Xiaotong Han, Elise Allee, Kathryn J Kotrla

**Affiliations:** 1 Division of Health Services Research, Department of Psychiatry, University of Arkansas for Medical Sciences, Little Rock, AR, USA; 2 Department of Biostatistics, College of Public Health, University of Arkansas for Medical Sciences, Little Rock, AR, USA; 3 Department of Psychiatry and Behavioral Sciences, College of Medicine, Texas A&M Health Science Center, College Station, TX, USA

## Abstract

**Background:**

Psychiatric and substance use problems are commonly found to be contributing factors to frequent Emergency Department (ED) use, yet little research has focused on the association between substance use and psychiatric comorbidity. This study assesses the association of a psychiatric comorbidity on (ED) use among patients with substance use disorders (SUDs).

**Methods:**

The study focuses on 6,865 patients who were diagnosed with SUDs in the ED of a large urban hospital in the southern United States from January 1994 – June 1998. Patients were grouped by type of substance use disorder. After examining frequency of visits by diagnosis, the sample was assigned to the following groups–alcohol dependence (ICD9 = 303), alcohol abuse (ICD9 = 305.0), cocaine dependence/abuse (ICD9 = 304.2, 305.6), and polysubstance/mixed use (ICD9 = 305.9). A patient was classified with psychiatric comorbidity if a psychiatric diagnosis appeared during any of the patient's visits. The following psychiatric diagnoses were included–schizophrenia/psychoses, bipolar disorder, depression, anxiety, and dementia (ICD-9 codes available upon request).

**Results:**

Patients with SUDs and psychiatric comorbidity had significantly higher mean number of ER visits (mean = 5.2 SD = 8.7) than SUD patients without psychiatric comorbidity (mean = 2.5, SD = 3.7). In logistic regressions predicting several categorizations of heavier use of the ED (either 4+, 8+, 12+, 16+, or 20+ visits over the span of the study) SUD patients with psychiatric comorbidity had adjusted odds ratios of 3.0 to 5.6 (reference group = patients with SUDs but no psychiatric comorbidity). This association was found across all substance use diagnostic categories studied, with the strongest relationship observed among patients with cocaine disorders or alcohol dependence.

**Conclusion:**

The results provide further support for the notion that the ED could and should serve as an important identification site for cost-effective intervention.

## Background

For some time, health services research has focused on the issue of frequent use of the ED. This growing literature finds that smaller subgroups of patients with repeat visits use disproportionate amounts of services. [1-4] From both clinical and policy perspectives, few would argue that frequent use of the ED is an optimal treatment approach. It is incumbent upon the field to identify the health and social issues driving frequent use of the ED and to identify suitable interventions to improve care and reduce the strain on scarce ED resources.

Research on frequent users of the ED find that they have fewer resourcesand higher rates of mortality and morbidity than non-frequent users. [5,6] Psychiatric and substance use problems are commonly found to be contributing factors to frequent ED use. [3,7-14] Little research, however, has focused on the association between substance use and psychiatric *comorbidity *and the frequency of ED use. A group of studies has found that comorbid substance use disorders were associated with increased ED use among persons with schizophrenia. [12,15,16]

A recent study by the current authors found that comorbid substance use disorders were significantly and substantially related to increased ED use across samples of ED users with a range of primary psychiatric disorders (e.g., schizophrenia, depression, anxiety, etc.). [17] The largest increases in ED use frequency were observed for patients with schizophrenia or dementia and a comorbidity of substance use disorders (generically defined). That study used data from the same hospital as the current study; however, the samples are mutually exclusive and there are no overlapping cases.

The current study is the first to our knowledge to examine the association of a comorbid psychiatric diagnosis to the frequency of ED visits of a cohort of patients who were discharged from an ED with a primary substance use disorder diagnosis. More specifically, the goal of the study was to document the association of psychiatric comorbidity to frequency of ED use among patients with *different *substance use disorders. The study authors' hypothesis was that psychiatric comorbidity would be associated with more frequent ED use across all substance use diagnostic groups studied. It is hoped that the identification of modifiable risk factors for frequent ED use could lead to the development of promising interventions in the future.

## Methods

### Data source and collection

The data used in the study originate from a large community hospital in the southern United States. The facility is a general medical/surgical hospital with a specialized psychiatric ED within the general ED. Data were gathered on every ED visit (total = 364,591) from January 1994 to June 1998. The hospital cares for approximately 60% of all county hospital ED patients. With the only level 1 trauma center in the area, the hospital handles most of the city's trauma and virtually all acutely ill indigent patients. The psychiatric emergency department is where law enforcement officers are instructed to take individuals needing psychiatric care, and was the only facility in the area equipped to handle involuntary indigent patients needing psychiatric evaluation during the study period. Patients presenting with psychiatric and/or substance use problems are directed to the psychiatric ED. All psychiatric diagnoses are made by psychiatrists.

Every psychiatric ED patient received a multi-axial assessment and diagnostic formulation. Diagnoses were made according to the Diagnostic and Statistical Manual of Mental Disorders III-R or IV. [18,19] The hospital's medical record allowed for the recording of four diagnoses per visit, including psychiatric, alcohol or substance related conditions, and medical conditions. All psychiatric diagnoses were made by the attending psychiatrists or by first or second year psychiatry residents who were directly supervised by the attending staff. During the entire study period there were three attending psychiatrists on staff, and the continuity of attending psychiatrists provided consistency in the diagnostic process. Because diagnosis in an emergency department setting may be difficult, [15] several safeguards were employed in the psychiatric ED to improve the quality of diagnosis. First, any suspicion of a medical condition causing the psychiatric presentation was evaluated by the internal medicine service to provide medical diagnosis and determine that the patients' presenting symptoms are due to psychiatric and not medical disorders. Second, the index of suspicion for substance abuse and substance induced psychiatric disorders was high for the presenting population, and a primary psychiatric diagnosis was not given if substance use is suspected as a primary etiologic factor.

In addition to the diagnostic information at each visit, demographic and patient entry and disposition data was recorded by emergency department nursing staff. Demographic information includes gender, race, and age. Data entry for the study period was supervised by a single individual who checked the accuracy of data input by comparison with the medical record. When the database was generated, DSM diagnoses were recorded as ICD-9 codes. The research was approved by the Institution Review Board at the University of Arkansas for Medical Sciences.

### The sample

Every patient with at least one primary discharge diagnosis of *any *substance use disorder from any area of the ED (medical, surgical, psychiatry) during the study span (n = 7,570) was included in the initial sample. This group made up 3.7% of the total number of unique patients using the ED across the span of the study (n = 203,114). These patients were then grouped by type of substance use disorder. After examining frequency of visits by diagnosis, the final sample (n = 6,865) was assigned to the following groups–alcohol dependence (ICD9 = 303), alcohol abuse (ICD9 = 305.0), cocaine dependence/abuse (ICD9 = 304.2, 305.6), and polysubstance/mixed use (ICD9 = 305.9). The alcohol dependence and abuse groups were not combined due to the large numbers of patients in each category. The cocaine abuse and dependence groups were combined due to the very small number of patients who received a cocaine dependence diagnosis. For the current study we excluded patients (n = 705) in less commonly presented diagnostic categories (e.g., opiate, hallucinogen, barbiturate, amphetamine, and marijuana use disorders to name several).

Because the diagnosis for a given patient could change from visit to visit, patients were placed in a diagnostic category based on the diagnosis received during a majority of visits. In the rare cases of "ties" in the number of visits falling in more than one diagnostic category, a grouping algorithm was used. If any tie involved "polysubstance use", the patient was placed in that category. Next, the following hierarchy of "severity", based on the clinical judgment of the authors, was imposed such that any remaining ties would be resolved by the patient being grouped in the more severe category–cocaine dependence/abuse, alcohol dependence, or alcohol abuse.

A patient was classified with psychiatric comorbidity if a psychiatric diagnosis appeared during any of the patient's visits. The following psychiatric diagnoses were included–schizophrenia/psychoses, bipolar disorder, depression, anxiety, and dementia (ICD-9 codes available upon request).

### Data Analyses

T-tests of group means were used to investigate differences in number of ED visits across our substance use categories by psychiatric comorbidity. Logistic regression analysis was used to test the predictive ability of the presence of psychiatric comorbidity on frequency of ED visits, controlling for age, race (Caucasian, African-American, Hispanic, other), and gender. Interaction effects were also tested between psychiatric comorbidity and age, race, and gender. Due to the large sample size, we used a conservative *p *value of .01. Separate logistic regression models were used for each substance use group. Five categories of "frequent ED use" were created: 4 or more visits (4+), 8 or more visits (8+), 12 or more visits (12 +), 16 or more visits (16+), and 20 or more visits (20+) across the 4.5-year span of the study. The rationale for using multiple categories was twofold: 1) The literature does not agree on what "frequent use" is, and providing a range of categories allows the data to be comparable to a broader range of previous work. 2) The categories allowed for "sensitivity analyses" to investigate if the predictive ability of the psychiatric comorbidity would be constant across frequency categories or if its strength as a predictor might level or drop-off after a certain number of visits. To arrive at these specific categories, the data on ED use were examined. The sample's mean number of visits across the span of the study was 2.9, with a standard deviation of 4.8. Based on these data, and the judgment of the clinician co-authors of the manuscript, it was decided that the categories would be based on a count of 4. The first category of frequent use (4+ visits) represents a value just beyond the mean as a lower bound. The next category (8+ visits) captures the number of visits corresponding to the first standard deviation. The remaining categories approximate the next standard deviations. This categorization also reflects the judgment of the clinician co-authors that it would be useful to have categories that correspond to 1+ mean visit per year of the study (4+ visits), 2+ mean visits per year of the study (8+ visits), up to 5+ means visits per year of the study (20+ visits). As well, this grouping corresponds closely to the categories used by one of the only other multi-year studies of repeat users of the ED by persons with psychiatric diagnoses. [16]

## Results

Patient demographic information is presented in Table 1. The sample was predominantly male (72.9%). African-Americans were more heavily represented in the polysubstance use, cocaine, and alcohol abuse groups; Caucasians were more represented in the alcohol dependence group. The most common presenting disorder was alcohol abuse (35.5%), followed by alcohol dependence (26.0%), cocaine (21.2%) and polysubstance use (17.4%) disorders. Patients with polysubstance use disorder were the most likely to also have been diagnosed with a psychiatric disorder (21.2%) in the ED. Patients with cocaine use disorders (14.3%) and alcohol dependence (14.1%) had similar rates of comorbid psychiatric disorders.

**Table 1:** Characteristics of ED Users with Primary Substance Use Disorder Diagnoses

**SUD Group**	**N (%)**	**Gender**		**Ethnicity**				**Age (years) (Mean/SD)**	**N (%) MH diagnosis**
		**N (%) Male**	**N (%) Female**	**N (%) African American**	**N (%) Hispanic**	**N (%) Caucasian**	**N (%) Other**		

**Total SUD**	6865 (100%)	5006 (72.9)	1859 (27.1)	2428 (35.4)	1677 (24.4)	2697 (39.3)	63 (0.9)	36.9 (14.2)	1002 (14.6)

**Polysubstance**	1191 (17.4%)	806 (67.7)	385 (32.3)	474 (39.8)	188 (15.8)	518 (43.5)	11 (0.9)	31.9 (13.5)	252 (21.2)

**Cocaine**	1453 (21.2%)	991 (68.2)	462 (31.8)	884 (60.8)	186 (12.8)	374 (25.7)	9 (0.6)	32.5 10.8)	207 (14.3)

**Alc. Dependence**	1785 (26.0%)	1333 (74.7)	452 (25.3)	442 (24.8)	561 (31.4)	761 (42.6)	21 (1.2)	39.8 16.3)	252 (14.1)

**Alcohol Abuse**	2436 (35.5%)	1876 (77.0)	560 (23.0)	628 (25.8)	742 (30.5)	1044 (42.9)	22 (0.9)	40.0 (13.3)	291 (12.0)

Note: SUD = substance use disorder; MH = mental health; Alc. = alcohol.

Overall, the group of primary substance use disorder patients without a recorded psychiatric comorbidity had a mean of 2.5 visits (SD = 3.7) over the study, while the patients with a psychiatric comorbidity had a mean of 5.2 visits (SD = 8.7; *t-test for group mean difference *significant at *p *< .001; *Kruskal Wallis test *significant at *p*= 0.02). Patients with psychiatric comorbidity had significantly more ED visits in every diagnostic category (data not shown) with similar mean values as noted above.

Adjusted odds ratios (OR) for frequent use of the ED are presented in Table 2. In multiple logistic regression analyses predicting frequent use of the ED, substance use patients with a comorbid psychiatric disorder were consistently more likely to be frequent users (reference groups = patients with a substance use disorder but no psychiatric disorder; covariates controlled for included age, race, and gender). For example, with the substance use diagnoses collapsed together into one group, the range of ORs for the comorbid patients ranged from 3.0 (*p *< .001) at 4+ visits to OR = 5.6 (*p *< .0001) for 20+ visits. The most substantial association of psychiatric comorbidity to frequency of ED use occurred in the cocaine group, whose ORs ranged from 3.5 (*p *< .001) at 4+ visits to 9.3 (*p *< .001) at 20+ visits. In terms of the relationships of the covariates to frequent ED use (data not shown), key findings were that males were significantly more likely to have more ED visits in all categories of ED use in all substance use groups except for cocaine, African-Americans were more likely to have more visits in all ED use categories and in all groups, and persons younger than 30 years of age were less likely to have frequent visits than persons over 45 in all ED use categories and in all substance use groups except cocaine. Interactions tested between psychiatric comorbidity and age, race, and gender were not statistically significant.

**Table 2:** Odds Ratios of Frequent Use of the ED for Substance Use Disorder Patients with Psychiatric Comorbidity vs. Those Without

SUD Group	**4 or more visits (CI)**	**8 or more visits (CI)**	**12 or more visits (CI)**	**16 or more visits (CI)**	**20 or more visits (CI)**
**Total SUD** N = 6865	3.0 (2.5, 3.4)^A^	4.0 (3.2, 4.9)^A^	5.0 (3.9, 6.5)^A^	5.0 (3.6, 7.0)^A^	5.6 (3.7, 8.4)^A^

**Polysubstance** N = 1191	3.9 (2.8, 5.5)^A^	4.7 (2.7, 8.2)^A^	3.4 (1.6, 7.5)^NS^	2.7 (0.9, 7.6)^NS^	3.0 (0.7, 12.7)^NS^

**Cocaine** N = 1453	3.5 (2.5, 4.9)^A^	6.4 (4.1, 10.1)^A^	6.8 (3.8, 12.1)^A^	9.1 (4.1, 20.0)^A^	9.3 (3.3, 25.7)^A^

**Alc. Dependence** N = 1785	2.9 (2.1, 3.9)^A^	4.2 (2.8, 6.3)^A^	5.2 (3.1, 8.6)^A^	5.7 (3.1, 10.7)^A^	6.1 (2.8, 13.5)^A^

**Alcohol Abuse** N = 2436	2.4 (1.8, 3.1)^A^	3.4 (2.4, 4.8)^A^	5.3 (3.5, 8.2)^A^	4.7 (2.8, 8.0)^A^	5.8 (3.1, 11.0)^A^

Note: SUD = Substance Use Disorder. CI = Confidence interval. Alc. = Alcohol. ^A ^= p < .001. ^NS ^= non-significant.

## Discussion

The data support the study's hypothesis that a comorbid psychiatric disorder among patients presenting to an ED with primary substance use disorders is associated with increased ED use. This association was found across all substance use diagnostic categories studied, with the strongest relationship observed among patients with cocaine disorders or alcohol dependence. The general trend across categories of frequency was for the association of psychiatric comorbidity to increase in magnitude, indicating that this combination of disorders might be an important risk factor for especially heavy use of the ED. It should be noted, however, that the 95% confidence intervals in the higher visit categories grew wide due to the smaller numbers of patients with higher numbers of visits, and thus, caution should be used in attributing robustness to the relationship to especially heavier use. Clinically speaking, the nonsignificant association of psychiatric comorbidity to higher categories of use among the polysubstance group was surprising. A dissimilar mixture of substance use patterns lumped together in this diagnostic category might have contributed to the weaker relationship. As well, this group contained the highest proportion of females and had the youngest mean age, and these factors might have also contributed to the weaker association with ED use. Further research is clearly needed to better understand service use and other outcomes associated with polysubstance use/psychiatric comorbidity.

Several limitations of this study should be noted. First, the data come from one facility, and may only be generalizable to urban community EDs in the southern United States. Further, the data come from an administrative database and the variables available for analysis were limited. Inclusion of measures such as severity of illness, income, and education would have been optimal. Also, it should be noted that no adjustment for risk to use ED services was available. Those that resided in the area longer had greater opportunity to use the ED and to be observed with a substance use condition than those who were more geographically mobile. It is plausible that persons with comorbid substance use disorders were more mobile during the study period than persons with psychiatric disorders alone, and if so, the observed relationships between comorbid substance use and ED frequency are likely underestimated. Most importantly, it should be noted that the data do not allow for a strict designation of causality. It is possible that the association between numbers of visits and comorbid psychiatric disorders could be opposite to the hypothesis–i.e., that a greater number of visits to the ED increases the probability that psychiatric disorders will be detected.

## Conclusion

Despite the study's limitations, and in light of its strengths (large, multi-year design with a closely validated administrative data collection process), the findings have important clinical and policy implications. If these findings are replicated in other ED settings, interventions should be developed to improve identification, referral, and appropriate treatment of substance use disorders in this comorbid population. Our data indicate that particular attention be paid to alcohol and cocaine use. Rockett and colleagues [22,23] have demonstrated the high unmet need for substance use treatment among ED patients, and the work of Cherpitel [24] suggests that the ED should be an important point for early identification and referral for treatment of substance use disorders. Cherpitel demonstrates that persons with alcohol problems make an alcohol-related ED visit relatively early in the pattern of alcohol-related health care use. [24] As such, the ED may provide a unique opportunity for referral and/or brief intervention.

Indeed, the literature has seen an increase in published reports of ED interventions to address both substance use and psychiatric disorders (though not together). A recent randomized study by Blow et al. [25] found several variations of brief interventions for at-risk drinking to be effective in reducing alcohol consumption among injured drinkers in an ED. Shumway et al. [26] tested a case management intervention in a 24-month randomized trial with 252 frequent ED users with psychosocial problems (e.g., substance abuse, psychiatric disorders, problems with housing or medical care). Case management (assessment, crisis intervention, supportive therapy, referrals, and linkage) was associated with significant reductions in ED use and costs compared to usual care. Another case management intervention for frequent users of the ED showed promise in linking patients with substance use disorders to needed services and reducing ED use. [27] A large case management intervention focusing on 607 ED patients with anxiety disorders found significant reductions in ED recidivism and costs at 6-months post-discharge from the ED. [28] A recent randomized trial of a behavioral/skills-building intervention found short-term decreases in ED use among older patients with schizophrenia. [29] Clearly, future research will continue to show that the ED can serve as an important identification site for cost-effective intervention.

## Competing interests

The authors declare that they have no competing interests.

## Authors' contributions

GC, GS, and KK conceived of the study, participated in its design, and helped draft the manuscript. KW helped conceive the study and contributed to the statistical design, analysis, and interpretation of the data. EA provided literature searches and helped draft the manuscript. XH performed the data analyses and helped draft the manuscript.

## Pre-publication history

The pre-publication history for this paper can be accessed here:


